# Transient Receptor Potential Is Essential for High Temperature Tolerance in Invasive *Bemisia tabaci* Middle East Asia Minor 1 Cryptic Species

**DOI:** 10.1371/journal.pone.0108428

**Published:** 2014-09-25

**Authors:** Zhi-Chuang Lü, Qian Li, Wan-Xue Liu, Fang-Hao Wan

**Affiliations:** 1 State Key Laboratory for Biology of Plant Diseases and Insect Pests, Institute of Plant Protection, Chinese Academy of Agricultural Sciences, Beijing, P. R. China; 2 Center for Management of Invasive Alien Species, Ministry of Agriculture, Beijing, China; Institute of Vegetables and Flowers, Chinese Academy of Agricultural Science, China

## Abstract

Temperature is an important factor in affecting population dynamics and diffusion distribution of organisms. Alien species can successfully invade and colonize to various temperature environments, and one of important reasons is that alien species have a strong resistance to stress temperature. Recently, researchers have focused on the mechanisms of temperature sensing to determine the sensing and regulation mechanisms of temperature adaptation. The transient receptor potential (TRP) is one of the key components of an organism’s temperature perception system. TRP plays important roles in perceiving temperature, such as avoiding high temperature, low temperature and choosing the optimum temperature. To assess high temperature sensation and the heat resistance role of the TRP gene, we used 3′ and 5′ rapid-amplification of cDNA ends to isolate the full-length cDNA sequence of the TRP gene from *Bemisia tabaci* (Gennadius) MEAM1 (Middle East Asia Minor 1), examined the mRNA expression profile under various temperature conditions, and identified the heat tolerance function. This is the first study to characterize the TRP gene of invasive *B. tabaci* MEAM1 (MEAM1 *BtTRP*). The full-length cDNA of MEAM1 *BtTRP* was 3871 bp, and the open reading frames of *BtTRP* was 3501 bp, encoding 1166 amino acids. Additionally, the *BtTRP* mRNA expression level was significantly increased at 35°C. Furthermore, compared with control treatments, the survival rate of *B. tabaci* MEAM1 adults was significantly decreased under high temperature stress conditions after feeding with dsRNA *BtTRP*. Collectively, these results showed that MEAM1 *BtTRP* is a key element in sensing high temperature and plays an essential role in *B. tabaci* MEAM1 heat tolerance ability. Our data improved our understanding of the mechanism of temperature sensation in *B. tabaci* MEAM1 at the molecular level and could contribute to the understanding of the thermal biology of *B. tabaci* MEAM1 within the context of global climate change.

## Introduction

The transient receptor potential (TRP) ion channel superfamily comprises a collection of cation channels that is conserved from worms to flies and humans [1], [2]. TRP channels, as membrane spanning proteins that regulate the flux of ions, play a central role in neurobiology. TRP channels are critical for sensing the external environment and are activated through various mechanisms and participate in virtually every sensory modality, such as temperature sensation, light sensation, and mechanistic sensation [3]. TRP channels have a profound impact on animal behavior. All animals have mechanisms to sense the temperature of their surroundings, and temperature sensation is mediated largely by direct activation of TRP ion channels [2], [4], [5]. TRP ion channels are activated by specific changes in temperature, acting as the molecular thermometers of the body, and TRP proteins enable sensory neurons to convey temperature information [6].

Insects are poikilothermic animals; therefore, they are extremely sensitive to changes in the temperature of the environment. However, although temperature sensation is critical for interaction with the environment, it has received relatively little attention from physiologists, [6], [7]. Flies have a simple neuronal architecture and genetic tractability, which make them an attractive animal model for studying the behavioral and molecular mechanisms underlying temperature sensation. Moreover, at present, most studies of TRP channel function in sensory physiology and animal behavior are focused on flies [3]. The need for thermo-sensation exists in all organisms, particularly for invasive species that are distributed widely and adapt to various climate regions. Therefore, we examined whether other non-model insects, such as invasive insects, also use TRP to sense temperature in order to gain insight into the fundamental biophysical mechanism concerning how temperature activates ion channels.

The whitefly *Bemisia tabaci* (Gennadius) (Hemiptera: Aleyrodidae) is a complex species, containing at least 30 morphologically indistinguishable cryptic species, such as Middle East-Asia Minor 1 (MEAM 1), Mediterranean cryptic species (MED) [8]. For our knowledge, the first major global invasion event is that of *B. tabaci* MEAM 1 cryptic species, and it is thought to have come from the Middle East-Asia Minor region [8], where its ability to tolerate exposure to extreme temperatures is central to its survival. MEAM 1 commenced sometime in the late 1980s principally via the trade in ornamentals [9], [10] (Broadbent et al., 1989; Cheek et al., 1994), from its origins to at least 54 countries [8]. MEAM 1 cryptic species is one of the most destructive invasive pests of field and glasshouse crops throughout the world [11], causing damage directly through feeding and indirectly through the transmission of plant pathogenic viruses, primarily begomoviruses [12]. For example, in Beijing and Turpan Xinjiang province, China, MEAM1 had caused great losses to vegetables and cottons, respectively, and the damage was up to 70% losses [13].

Invasive species usually have great potential to adapt to various environmental temperatures [14]–[15]. Previous studies have suggested that the ability of *B. tabaci* MEAM1 to resist heat may be one of the mechanisms that potentially underlies its invasive traits [15]–[17]. Lü and Wan [18] found that *hsp23* and *hsp70* plays a key role in heat tolerance in *B. tabaci* MEAM1 females. However, it remains unclear how the information of surrounding temperature transfers to the insect’s neural network and stimulates the expression of *hsp23* and *hsp70* to increase the heat resistance ability of *B. tabaci* MEAM1 adults.

To assess the high temperature sensation mechanism and heat resistance role of the TRP gene, the following aspects were explored. First, we cloned the full cDNA sequence of the TRP gene of *B. tabaci* MEAM1 (MEAM1 *BtTRP*). Second, we examined the mRNA expression profile of the MEAM1 *BtTRP* gene under various temperature conditions by quantitative real-time PCR. Third, we identified the function of the MEAM1 *BtTRP* gene under high temperature stress using the feeding dsRNA method. The aim was to reveal the characterization of the MEAM1 *BtTRP* gene and its temperature sensation role in *B. tabaci* MEAM1. This information could contribute to the understanding of the thermal biology of *B. tabaci* MEAM1 species within the context of global climate change.

## Materials and Methods

### Insects and host plants

*B. tabaci* MEAM1was reared on cotton plants, *Gossypium hirsutum* (L.) (var. Simian No. 3), in a glasshouse at 20–34°C, 50–60% RH and a natural photoperiod (39°55′ N, 116°20′ E). The plants were individually grown in 9-cm-diameter pots under the same conditions as the whitefly.

### Temperature stress treatments

To determine the role of the *BtTRP* gene in the ability to survive temperature stress exposure, we analyzed the relationship between thermotolerance and *BtTRP* mRNA expression. The thermotolerance test was conducted using the method described in [17], [19]. Because Bowler and Terblanche observed that adult age is associated with different responses to temperature stress [20], we standardized adult age using only newly emerged whitefly adults that were younger than 3 hours. One hundred females or males were placed together in a 1.5-mL centrifuge tube. To confirm that all of the whitefly adults underwent temperature stress, the tube was covered with cotton along 1/4 tube length from the top of the tube. The number of adults chosen was based on preliminary experiments showing that 100 adults enabled sufficient total RNA to be extracted for reverse transcription. The whiteflies inside the tubes were exposed to 1, 3, 5, 7, 9, 11, 13, 15, 17, 19, 21 and 23°C for 1 h in a constant environment (K6-cc-NR; Huber Kältemaschinenbau GmbH, Offenburg, Germany) and 29, 31, 33, 35, 37, 39, 41, 43 and 45°C for 1 h in a water bath (CC-106A; Huber Kältemaschinenbau GmbH, Offenburg, Germany). The treatment temperatures were selected based on our previous study [21]. The selected length of exposure was based on preliminary experiments indicating that a 1-hour exposure was sufficient to induce a measurable stress response in whitefly adults. Adults maintained at 26°C were used as untreated controls. Following stress exposure, the samples were frozen immediately with liquid nitrogen and then stored at −80°C until RNA extraction. Each treatment had five replicates.

### Rapid amplification of cDNA ends (RACE) and sequence analysis of full-length *BtTRP* cDNA

Total RNA was isolated using the RNeasy Mini Kit (Qiagen, Valencia, CA, USA), and 2 µg of RNA was used to generate cDNA using the oligo(dT)_15_ primer according to the instructions provided with the Reverse Transcription System (Invitrogen Life Technologies, Burlington, ON, Canada). Degenerate primers (Table 1) were used to amplify partial segments of the *BtTRP* gene. Next, 5′ and 3′ RACE was performed to obtain full-length cDNAs according to the manufacturer’s instructions (Rapid Amplification of cDNA Ends System, version 2.0; Invitrogen, Carlsbad, CA, USA) using gene-specific primers corresponding to GSP1 and GSP2 (Table 1). To ensure that the 5′ and 3′ fragments were derived from the same gene, specific primer sets flanking the open reading frames (ORFs) were designed and used to amplify the full-length cDNAs.

**Table 1:** Primer sequences used for cDNA cloning, real-time quantitative PCR and dsRNA synthesis.

Gene	Primer sequence (5′→3′)	Fragment length (bp)
PCR	CCACCATTCAACCTATCAC	759
	AACATCGTCTTTGCCTTC	
3′RACE-GSP1	ACACCGAGCGTGGACAAAGAGGA	1178
3′RACE-GSP2	CTATTGCCGCCAAGAAAGCATCCAG	
5′RACE-GSP1	TTGGCGGCAATAGCGTTCCAGTCC	2509
5′RACE-GSP2	CTCTTTGTCCACGCTCGGTGTCTT	
Real-time quantitative PCR		
NADH-F	ATAGTTGGCTGTAGAACCAGAGTG	96
NADH-R	ACACGAAGGGAAGAGCACATA	
*BtTRP*-F	GAAGACACCGAGCGTGGACAAAG	217
*BtTRP*-R	GGCAATAGCGTTCCAGTCCTTTT	
dsRNA synthesis primers		
	TAATACGACTCACTATAGGGAGACCAC GAAGACACCGAGCGTGGACAAAG	244
	TAATACGACTCACTATAGGGAGACCAC GGCAATAGCGTTCCAGTCCTTTT	

Primer sequences (no underline) are shown for PCR amplification, rapid amplification of cDNA ends (RACE) of *BtTRP* gene, and the relative quantification real-time PCR for detecting *BtTRP* mRNA expression patterns; primer sequences plus T7 promoter sequences (underlined) are shown for production of dsRNA transcription templates.

Based on the transcriptome information of *B. tabaci* [22]–[24], the full length *BtTRP* cDNA from *B. tabaci* MEAM1 was used as the query sequence to search for other insect TRP genes in the GenBank database using the BLAST software available on the NCBI website (http://www.ncbi.nlm.gov/BLAST/). Sequence alignment and identity analyses were performed using DNAMAN (version 5.0; Lynnon BioSoft, Quebec, Canada). The ORFs were identified using ORF Finder (http://www.ncbi.nlm.nih.gov/gorf/gorf.html). The amino acid sequences and molecular weight of the proteins were calculated using DNASTAR.

To evaluate the molecular evolutionary relationship of TRP from various insects, phylogenetic trees were constructed based on their protein sequences. Sequence homology searches were performed using BLAST, and all of the sequences were retrieved from GenBank using Blast-N and Blast-X. The retrieved sequences were aligned using the multiple alignment tool of the ClustalX program. Gaps and missing data were excluded from the data analysis. MEGA 5.1 was used to perform the tree calculations. The tree constructions were performed by using the Maximum Likelihood method based on the poisson correction mode. Support for the nodes was assessed as a proportion of 1000 bootstrap replicates to derive the confidence values of the phylogeny analysis.

### Real-time quantitative PCR

Total RNA from the samples was extracted using the RNeasy Mini Kit (Qiagen), and the RNase-Free Set (Qiagen) was used to remove genomic DNA. The quantity and quality of the RNA were assessed via spectrophotometry (Beckman Du 650 spectrophotometer, Fullerton, CA, USA), and the A260/A280 ratios were typically above 1.8. The RNA quality was also evaluated via 1% agarose gel electrophoresis. According to the manufacturer’s instructions, 2 µg of total RNA was used to synthesize cDNAs using the SuperScript III Reverse Transcriptase Kit (Invitrogen Life Technologies). The cDNA was stored at −80°C until further analysis.

The mRNA expression levels of *BtTRP* following exposure to various temperature stresses were examined via comparative quantitative real-time PCR analysis. The sequences of the primers are listed in Table 1. The reactions were performed using an iQ 5 Real-Time PCR Detection System (BioRad, Foster City, CA, USA). The amplification volume was 20 µL, including 0.5 µL of the forward primer (10 mM/µL), 0.5 µL of the reverse primer (10 mM/µL), 10.0 µL of 2×TransStart Green qPCR SuperMix, 0.4 µL of Rox, 1.0 µL of the cDNA sample and 7.6 µL of ultra-pure water. The PCR cycle conditions were as follows: 94°C for 5 min, followed by 40 cycles of amplification consisting of 94°C for 30 s, 58°C for 30 s and 72°C for 1 min, and then 72°C for 10 min. After the amplification phase, a dissociation curve was generated to ensure that there was only one product. A control without any template was included in all of the batches. The amplification efficiency was validated by constructing a standard curve using five serial dilutions of cDNA. The data were analyzed based on the C_p_ method according to the mathematical model of [25], simplified to 2^△△Ct^ as follows:
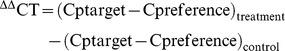



A 26°C sample was used as a control, and nicotinamide adenine dinucleotide (NADH) was used as the reference gene based on our preliminary experiments, which revealed that NADH was stably expressed under various temperature stress conditions (unpublished data). The relative expression level of *BtTRP* mRNA was defined as the fold-change normalized to the amount of NADH. Each sample was assessed in triplicate.

### Production of dsRNA transcription templates and synthesis of dsRNA

*BtTRP* transcription templates were produced from total whitefly cDNA using gene-specific primers containing a T7 promoter sequence; the T7 primer was as described previously [26]. Amplification reactions were conducted in 50 µL containing 38.0 µL of ddH_2_O, 5.0 µL of 10×buffer, 1.0 µL of dNTPs (10 mM for each nucleotide), 2.0 µL of forward primer (10 mM/µL), 2.0 µL of reverse primer (10 mM/µL), 1.0 µL of cDNA template and 1.0 µL of Taq DNA Polymerase (5 UµL^−1^; TransStart). The PCR cycling conditions were as follows: 94°C for 5 min, followed by 35 cycles of 94°C for 30 s, 60°C for 30 s and 72°C for 30 s, and a final extension step of 72°C for 10 min. The amplification of PCR products was confirmed by separation on 1.5% agarose gels and visualized by staining with ethidium bromide under UV light. The PCR products were purified using a Qiaquick PCR purification kit (Qiagen, Inc., Hilden, Germany) according to the manufacturer’s instructions. The PCR products were stored at −80°C prior to the synthesis of dsRNA.

DsRNA was synthesized using the MEGAscript RNAi Kit (Ambion, Austin, TX, USA), and 1 µg of PCR product was used as the transcription template. dsRNA was resuspended in RNase-free water. dsRNA was analyzed by agarose gel electrophoresis and quantified spectrophotometrically. The dsRNA was stored at −80°C prior to further use.

### Feeding of dsRNA and detection

Newly emerged whitefly adults were fed a diet containing dsRNA diluted to 0.3∼0.5 µg/µL in a 10% w/v RNase-free sucrose solution. Feeding was performed using the parafilm clip nutrient solution method [18], [27]. The parafilm was pre-treated with 0.1% diethyl pyrocarbonate (DEPC) solution to remove any RNases, and then RNase-free water was used to clean the DEPC from the parafilm. Two hundred newly emerged whitefly adults were collected and placed into a glass tube (3 cm in diameter, 8 cm in height). The tube opening was then covered with two layers of parafilm, and 200∼250 µL of dsRNA solution was injected into the gap between the two layers. The other end of the tube was covered with gauze to enable ventilation. The tube was then wrapped with black plastic paper leaving the parafilm enclosed end exposed to light. This process encouraged the adults to move toward the diet and feed. Each tube was then placed in a constant environment room for 3 h at 26±0.2°C. At 3 h, some of the samples were next placed immediately into liquid nitrogen to be frozen and then were stored at −80°C until RNA extraction. The remaining flies were exposed to 45±0.2°C in a water bath for 1 h after which they were then placed into another constant environment room at 26±0.2°C for 1 h; the number of live whiteflies was then counted. A temperature of 45°C was selected based on preliminary experiments showing that this temperature was the discrimination point for whitefly heat tolerance. The treated control comprised whiteflies fed 10% w/v RNase-free sucrose solution only, and the untreated control was composed of whiteflies fed nothing. Each treatment had five replicates.

*BtTRP* mRNA expression after dsRNA feeding was analyzed by comparative quantification real-time PCR. Additionally, the real-time PCR protocol followed the same method described above (“Real-time quantitative PCR”).

### Statistical analysis

Statistical analyses were conducted using SPSS package (version 13). Data were first tested for normality using the Kolmogorov-Smirnov test; the data were then log transformed to ensure that they were normally distributed. The effects of temperature on *BtTRP* gene expression and survival rate after feeding with the dsRNA mixture were analyzed by one-way ANOVA. The means were separated using LSD α = 0.05. The target gene expression after feeding with the dsRNA mixture was analyzed by independent T-test. The results were expressed as the means ± standard error (mean ± SEM). The differences were considered significant when the P-values were less than or equal to 0.05.

## Results

### cDNA sequence analysis of *BtTRP* (characterization and homology, phylogenetic analysis)

**Figure 1:**
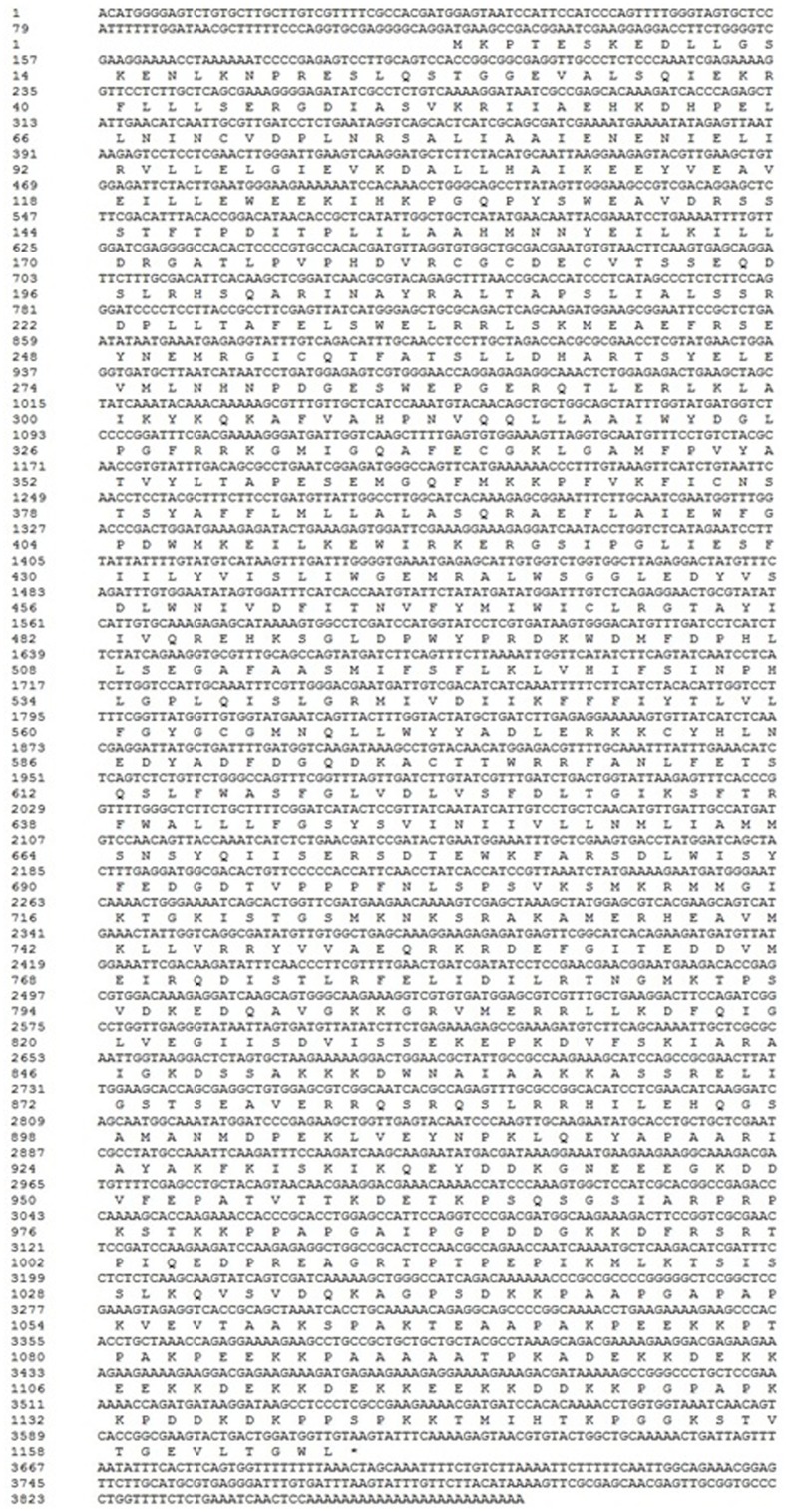
The full-length cDNA sequence of *Bemisia tabaci* MEAM1 *BtTRP* and its deduced amino acid sequence. The full-length cDNA of MEAM1 *BtTRP* is 3871 bp, and the ORF is 3501 bp, which encodes a polypeptide of 1166 amino acids. The underlined amino acid positions of 369–391, 420–442, 457–479, 507–529, 551–573 and 638–660 indicate the transmembrane structural positions of S1, S2, S3, S4, S5 and S6, respectively. The highly conserved TRP gene structural domain, EWKFAR, was located between amino acids 675–700. The motif protein was located between amino acids 69 and 173. The asterisk indicates the translational termination codon.

The full-length cDNA of MEAM1 *BtTR*P (KM280572) is 3871 bp, including a 5′-terminal UTR of 118 b (the position of 1–118), a 3′-terminal UTR of 252 bp (the position of 3620–3871) containing a poly (A) tail, and an ORF of 3501 bp (the position of 119–3619) encoding a polypeptide of 1166 amino acids with a predicted molecular mass of 132.0 kDa. One highly conserved TRP gene structural domain, EWKFAR, was identified and was located between amino acids 675–700. The motif protein was located between amino acids 69 and 173 by using on line software (http://www.ncbi.nlm.nih.gov/Stucture/bwrpsb/bwrpsb.Cgi) (Fig. 1).

**Figure 2:**
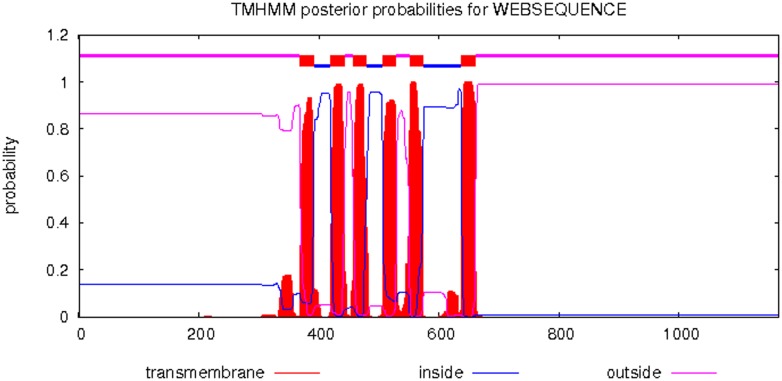
Transmembrane structure prediction of the *BtTRP* protein in *Bemisia tabaci* MEAM1. Six transmembrane structures were found in MEAM1 *BtTRP*, and one pore ring between the fifth and sixth transmembranes. The amino acid number of each transmembrane structural domain (from S1 to S6) was 23. The transmembrane structural positions of S1, S2, S3, S4, S5 and S6 was located at the amino acid positions of 369–391, 420–442, 457–479, 507–529, 551–573 and 638–660, respectively.

The transmembrane helices in TRP protein were predicted by using on line software TMHMM Server v. 2.0 (http://www.cbs.dtu.dk/services/TMHMM-2.0/). As showed in Fig. 2, six transmembrane structures were found in MEAM1 *BtTRP*, and one pore ring between the fifth and sixth transmembranes. The amino acid number of each transmembrane structural domain (from S1 to S6) was 23. The amino acid positions of 369–391, 420–442, 457–479, 507–529, 551–573 and 638–660 (Fig. 1) indicated the transmembrane structure positions of S1, S2, S3, S4, S5 and S6, respectively.

**Figure 3:**
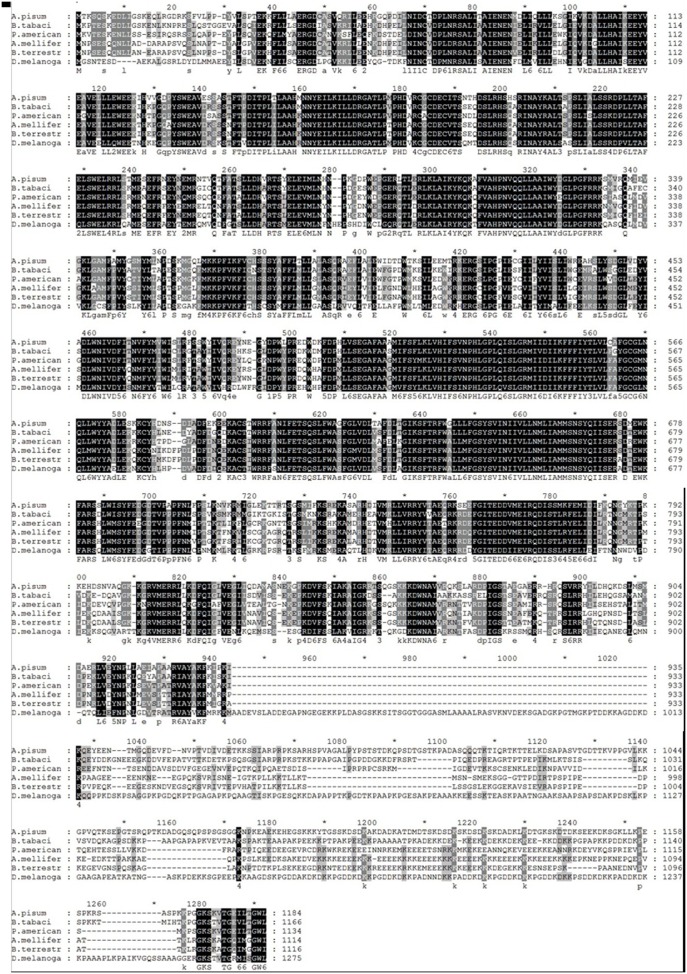
Alignment of TRP protein from *Bemisia tabaci* MEAM1 and other insects. It was showed that the deduced amino acid sequence of *BtTRP* is highly conserved when compared with previously identified TRP amino acid sequences. *A. pisum*: *Acyrthosiphon pisum* TRP (XP_003240303.1); *A. mellifera*: *Apis mellifera* TRP (XP_001120503.2); *B. terrestris*: *Bombus terrestris* TRP (XP_003402180.1); *P. Americana*: *Periplaneta Americana* TRP (AGG86916.1); *D. melanogaster*: *Drosophila melanogaster* TRP (AAA28976.1); *B. tabaci*: *Bemisia tabaci* Middle East-Asia (MEAM1).

**Figure 4:**
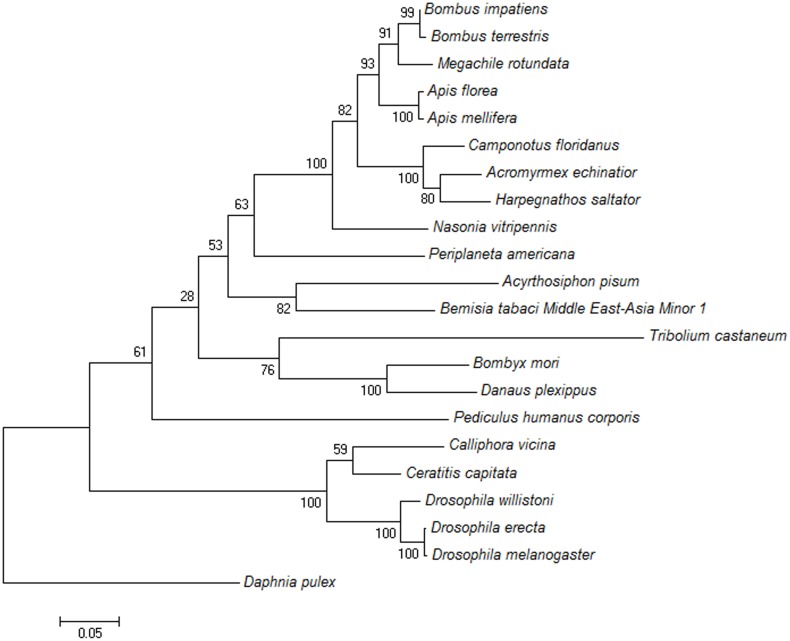
A phylogenetic tree based on the known amino acid sequences of TRP genes. The phylogenetic tree was generated via Maximum Likelihood method based on the poisson correction mode, and this tree was used to determine the relationships between different insects. The numbers above the branches indicate the percentages of bootstrap replicates in which each species was grouped together. The scale bar indicates the number of substitutions per site for each unit branch length. The bootstrap values of 1000 replicates are displayed for each branch. TRP from insects of the same order were clustered into the same group, which is consistent with traditional taxonomy. *Bombus terrestris* (XP_003402180.1); *Bombus impatiens* (XP_003489572.1); *Megachile rotundata* (XP_003705368.1); *Apis mellifera* (XP_003489572.1); *Apis florea* (XP_003696373.1); *Camponotus floridanus* (EFN73452.1); *Harpegnathos saltator* (EFN82264.1); *Acromyrmex echinatior* (EGI64238.1); *Nasonia vitripennis* (XP_001605329.1); *Periplaneta Americana* (AGG86916.1); *Acyrthosiphon pisum* (XP_003240303.1); *Ceratitis capitata* (XP_004536899.1); *Calliphora vicina* (CAB02410.1); *Drosophila willistoni* (XP_002073367.1); *Drosophila melanogaster* (NP_476768.1); *Drosophila erecta* (XP_001981285.1); *Pediculus humanus corporis* (XP_002423380.1); *Tribolium castaneum* (XP_968670.2); *Danaus plexippus* (EHJ65374.1); *Bombyx mori* (XP_004922653.1); *Daphnia pulex* (EFX85740.1).

In addition, homology analysis of Fig. 3 revealed that, compared with previously identified TRP genes, the identity of the deduced amino acid sequence of *BtTRP* is highly conserved, with highly conserved TRP gene structural domain, EWKFAR, locating between amino acids 675–700 (Fig. 1). Furthermore, as shown in Fig. 4, TRP from insects of the same order were clustered into the same group, such as *Bombus terrestris, Bombus impatiens, Megachile rotundata, Apis mellifera, Apis florea, Camponotus floridanus*, *Harpegnathos saltator*, *Acromyrmex echinatior* and *Nasonia vitripennis* clustered into Hymenoptera, *Ceratitis capitata*, *Calliphora vicina, Drosophila willistoni*, *Drosophila melanogaster* and *Drosophila erecta* clustered into Hymenoptera, etc. The result is consistent with traditional taxonomy.

### *BtTRP* mRNA expression pattern in *B. tabaci* MEAM1 under heat shock conditions

**Figure 5:**
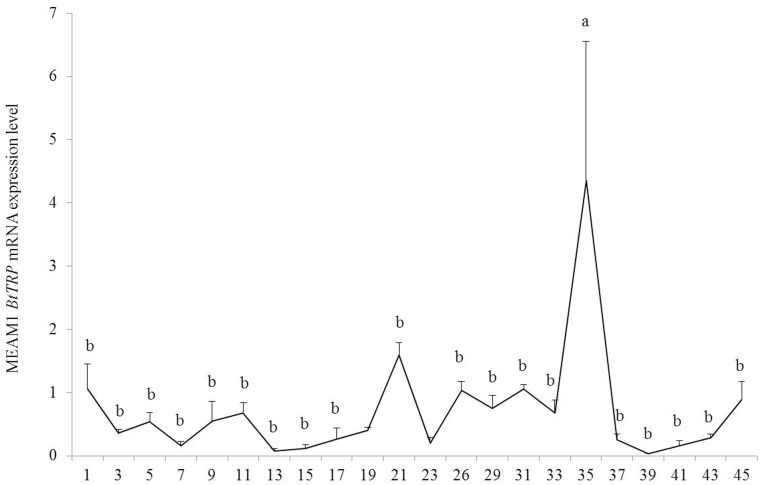
*Bemisia tabaci* MEAM1 *BtTRP* mRNA expression following a 1-h treatment at the temperature indicated. The results showed that temperature had a significant effect on *BtTRP* mRNA expression, with different mRNA expression level following different temperature stress, and the *BtTRP* mRNA expression level significantly increased at 35°C. The results are expressed as the means ± SEM. The means over the bars followed by different lowercase letters are significantly different at *P*≤0.05.

The sequences of all the primers of the relative quantification real-time PCR for detecting *BtTRP* mRNA expression patterns are listed in Table 1. Temperature had a significant effect on *BtTRP* mRNA expression (F_21,87_ = 3.345, P<0.05) (Fig. 5). Compared with other stress temperatures, the *BtTRP* mRNA expression level significantly increased at 35°C, and it was not significant difference under other stress temperature conditions (Fig. 5).

### The role of the *BtTRP* gene during heat shock treatment

**Figure 6:**
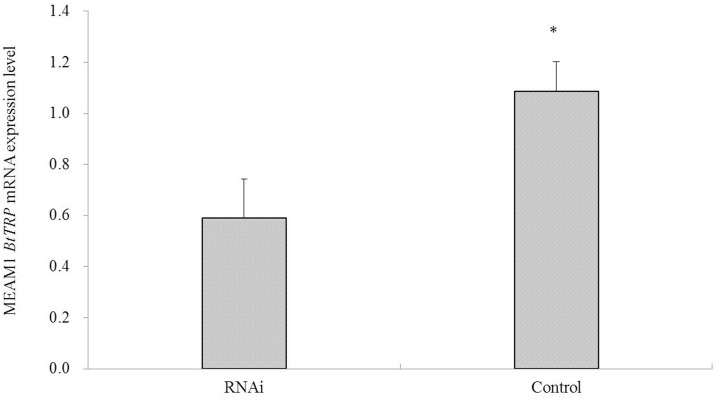
The effect of dsRNA treatment on the mRNA expression of *Bemisia tabaci* MEAM1 *BtTRP*. It was showed that, compared with the controls, *BtTRP* mRNA expression was significantly decreased in *B. tabaci* MEAM1 after feeding with dsRNA for 3 h. The results are expressed as the means ± SEM. The means over the bars followed by “*” are significantly different at *P*≤0.05.

The sequences of the primers of comparative quantification real-time PCR for detecting mRNA expression after feeding with dsRNA and production of dsRNA transcription templates are listed in Table 1. Compared with the controls, *BtTRP* mRNA expression was significantly decreased in half in *B. tabaci* MEAM1 after feeding with dsRNA for 3 h (F = 2.765, *P*<0.05) (Fig. 6).

**Figure 7:**
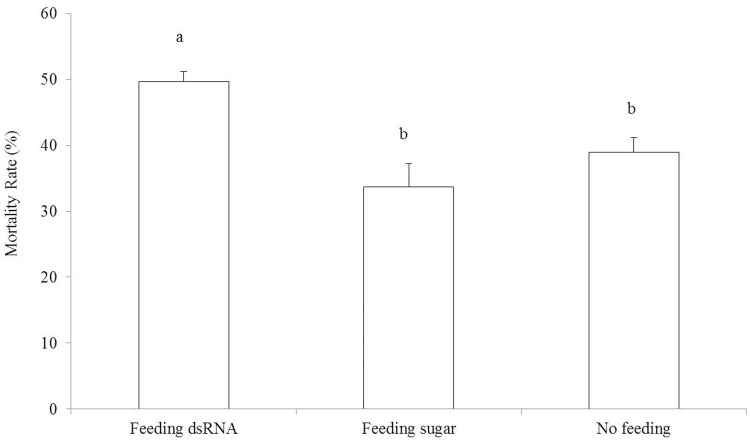
The effect of dsRNA *BtTRP* on the heat tolerant ability of *Bemisia tabaci* MEAM1 adults. The results showed that, compared with the control treatments, the survival rate of whitefly adults was significantly decreased at 45°C for 1 h after feeding with *BtTRP* dsRNA. It suggested that *BtTRP* was one key factors influencing adult heat tolerance. The results are expressed as the means ± SEM. The means over the bars followed by different lowercase letters or capital letters are significantly different at *P*≤0.05.

Furthermore, as showed in Fig. 7, compared with the control treatments, the survival rate of whitefly adults was significantly decreased at 45°C for 1 h after feeding with *BtTRP* dsRNA (F_2,12_ = 9.806; *P*<0.05). The mortality rate of feeding dsRNA, feeding sugar and no feeding was 49.6%, 33.7% and 38.9%, respectively. The results showed that *BtTRP* influenced adult heat tolerance.

## Discussion

The TRP channel gene has been previously sequenced in the following model insects: *Drosophila melanogaster* (Diptera), *Bombyx mori* (Lepidoptera), *Tribolium castaneum* (Coleoptera), *Apis mellifera* (Hymenoptera), *Nasonia vitripennis* (Hymenoptera), and *Pediculus humanus* (Phthiraptera) [28]. However, the MEAM1 *BtTRP* gene full-length cDNA sequence generated in the present study represents the first TRP sequence cloned from an invasive non-model insect. The N-terminal anchoring protein binding site of the MEAM1 *BtTRP* gene was located at amino acid positions 69 to 173. The interaction between the anchored proteins and TRP channel might inhibit the release of intracellular Ca^2+^, connecting the TRP channel and cytoskeleton and forming heteromerics. Generally, the C-terminal conserved structure of the TRP gene is composed of 23–25 amino acids [2], [29]–[31]. Interestingly, 30 amino acids in the C-terminal conserved structure of the MEAM1 *BtTRP* gene are located at amino acid positions 1096 to 1125. The TRP superfamily of cation channels shares six common transmembrane domains and permeability to cations [2], [28]. Similarly, for the transmembrane domain analysis of amino acids using TMHMN online software, we found six α transmembrane domains in MEAM1 *BtTRP*, a finding that is consistent with the results of other studies, but we did not find the amino acids responsible for detecting a voltage change in the fourth transmembrane domain [2]. The transmembrane structural analysis of TRP has indicated that the N- and C-termini are located within cells [29]–[31]. Intriguingly, in the present study, we found that the N- and C-termini were located inside the cell membrane. Matsuura et al. [28] reported that both evolutionary conservation and changes occurred in insect TRP channels and that the rate of evolutionary change was accelerated in this family. In the present study, the phylogenetic trees showed that TRP from insects of the same order were clustered into the same group, a finding that was consistent with traditional taxonomy. Invasive *B. tabaci* MEAM1 can adapt to various climate regions and might possess a unique temperature sensing mechanism. Thus, further examination is needed to determine whether unique MEAM1 *BtTRP* gene features, including a longer C-terminal conserved structure and the N- and C-termini inside the cell membrane, are related to its temperature sensing characteristics.

TRP channels have crucial functions for various sensory modalities. Many TRP channels are activated by various stimuli and function as primary signal integrators. TRP channels are expressed and function in various organisms, such as nematodes, fruit flies, fish, mice and humans [28]. DmTRP has been shown to be activated increases in temperature [29], [32]. Additionally, Matsuura et al. [28] showed that AmTRPA5 expressed in HEK293 cells was not activated by temperature fluctuations. In the present study, the mRNA expression level of the MEAM1 *BtTRP* gene was significantly increased at 35°C. For the same high temperature stress conditions, our previous study showed that the onset temperatures (T_on_s) of the synthesis of *hsp20*, *hsp70* and *hsp90* were 35°C, 39°C and 35°C, respectively. Additionally, the mRNA expression maximum temperatures (T_max_s) of *hsp20*, *hsp70* and *hsp90* were 39°C, 41°C and 39°C, respectively [21]. These results showed that the T_on_s of *hsp70* expression in *B. tabaci* MEAM1 were generally 4°C higher than that of MEAM1 *BtTRP* mRNA expression, and the T_on_s of *hsp20* and *hsp90* expression were the same as the temperature of the significant MEAM1 *BtTRP* expression level increase. The T_max_s of the expression of the three hsp genes were generally 4–6°C higher that of the MEAM1 *BtTRP* mRNA expression peak in *B. tabaci* MEAM1. The mRNA expression characteristics of the *BtTRP* and *hsp* genes suggested that the *BtTRP* gene sensed the environment temperature change and transferred the information to *B. tabaci* MEAM1, activating a series of physiological actions, such as induction of the expression of heat shock protein genes, to improve the organism’s heat tolerance ability.

To date, the physiological functions of TRP channels have been exclusively characterized in fruit flies and mice [28]. Thermo-sensitive TRP channels are specific TRP channels that are activated by an increase or a decrease in temperature [2], [33], [34]. All thermo-sensitive TRP channels belong to the TRPA subfamily in fruit flies [35]–[38], which consists of TRPA1, Painless (Pain), Pyrexia (Pyx) and Waterwitch (Wtrw) [3]. *Drosophila* Pain and Pyx function as thermosensors, responding to different “hot” temperatures, and the temperature thresholds for activating Pain and Pyx were shown to be 42.6 and 37.5–40°C, respectively [35]–[36], [39], suggesting that they were direct sensors of noxious heat [3]. Additionally, TRPA1 and Pain contributed to avoidance of 46°C noxious heat in *Drosophila* [40]. DmTRPA1 was found to be necessary for *Drosophila* larvae to discriminate between 18°C and 24°C [41]. Sato et al. showed that *BmTrpA1* was activated at temperatures above ∼21°C in *Bombyx mori* [42]. Furthermore, Rosenzweig et al. showed that RNA interference (RNAi)-mediated knockdown of dTRPA1 caused warm avoidance defects in *Drosophila* larvae, suggesting that dTRPA1 was essential for thermotaxis in *Drosophila* [37]. In recent years, RNAi technology has been successfully applied to the study of gene silencing in whiteflies [18], [26]. Our previous work indicated that feeding with dsRNA could lead to the inhibition of target gene mRNA expression [18] because whiteflies have a cross-membrane transport mechanism. Thus, we used the feeding dsRNA method to identify the function of the MEAM1 *BtTRP* gene under high temperature conditions. The present study showed that, compared with control treatments, feeding with MEAM1 *BtTRP* dsRNA significantly decreased survival rates, supporting the idea that MEAM1 *BtTRP* plays an essential role in the heat tolerance of *B. tabaci* MEAM1. According to previous studies that all thermo-sensitive TRP channels belong to the TRPA subfamily in fruit flies [3], [35]–[37], we suggest that MEAM1 *BtTRP* belongs to the TRPA subfamily because the MEAM1 *BtTRP* channel contributes to increased high temperature stress tolerance.

### Conclusions

In summary, the present study was the first to characterize *BtTRP* in invasive *B. tabaci* MEAM1 and the mRNA expression profile during different temperature stress conditions and under high temperature stress in a physiological model. The result of feeding *BtTRP* dsRNA showed that MEAM1 *BtTRP* is a key element in sensing high temperature and plays a key role in *B. tabaci* MEAM1 heat tolerance ability. Our data improved our understanding of the mechanism of temperature sensation in *B. tabaci* MEAM1 at the molecular level. However, the precise physiological function of MEAM1 *BtTRP* under low temperature conditions warrants further investigation. How the temperature of the environment transfers into the body needs to be investigated further using electrophysiological methods.

## References

[1] Ramsey IS, Delling M, Clapham DE (2006) An introduction to TRP channels. Annu Rev Physiol 68: 619–647. 16460286 10.1146/annurev.physiol.68.040204.100431

[2] Venkatachalam K, Montell C (2007) TRP channels. Annu Rev Biochem 76: 387–417. 17579562 10.1146/annurev.biochem.75.103004.142819 PMC4196875

[3] Fowler MA, Montell C (2013) *Drosophila* TRP channels and animal behavior. Life Sci 92: 394–403. 22877650 10.1016/j.lfs.2012.07.029 PMC3524398

[4] Bandel M, Macpherson LJ, Patapoutian A (2007) From chills to chilis: mechanisms for thermosensation and chemesthesis via thermoTRPs. Curr Opin Neurobiol 17: 490–497. 17706410 10.1016/j.conb.2007.07.014 PMC2080617

[5] Caterina MJ (2007) Transient receptor potential ion channels as participants in thermosensation and thermoregulation. Am J Physio - Reg I 292: 64–76. 10.1152/ajpregu.00446.2006 16973931

[6] Patapoutian A (2005) TRP channels and thermosensation. Chem Senses 30 suppl 1: i193–i194. 15738110 10.1093/chemse/bjh180

[7] Hensel H (1981) Thermoreception and temperature regulation. Monogr Physiol Soc 38: 1–321. 6820811

[8] De Barro PJ, Liu SS, Boykin LM, Dinsdale AB (2011) *Bemisia tabaci*: a statement of species status. Annu Rev Entomol 56: 1–19. 20690829 10.1146/annurev-ento-112408-085504

[9] Broadbent AB, Foottit RG, Murphy GD (1989) Sweetpotato whitefly *Bemisia tabaci* (Gennadius) (Homoptera:Aleyrodidae), a potential insect pest in Canada. Can Entomol 121: 1027–28.

[10] Cheek S, Macdonald O (1994) Statutory controls to prevent the establishment of *Bemisia tabaci* in the United Kingdom. Pestic Sci 42: 135–42.

[11] Oliveira MRV, Henneberry TJ, Anderson P (2001) History, current status, and collaborative research projects for *Bemisia tabaci* . Crop Prot 20: 709–723.

[12] Jones DR (2003) Plant viruses transmitted by whiteflies. Eur J Plant Pathol 109: 195–219.

[13] Luo C, Zhang ZL (2000) Thanking about Bemisia tabaci outbreaks. Beijing Sci 18: 4–13.

[14] Lee CE (2002) Evolutionary genetics of invasive species. Trends Ecol Evol 17: 386–391.

[15] Wan FH, Zhang GF, Liu SS, Luo C, Chu D, et al (2009) Invasive mechanism and management strategy of *Bemisia tabaci* (Gennadius) biotype B: Progress report of 973 Program on invasive alien species in China. Chinese Sci Ser C 52: 88–95. 10.1007/s11427-008-0135-4 19152088

[16] Cui XH, Wan FH, Xie M, Liu TX (2008) Effects of heat shock on survival and reproduction of two whitefly species, *Trialeurodes vaporariorum* and *Bemisia tabaci* biotype B. J Insect Sci. 24: 1–10.

[17] Yu H, Wan FH, Guo JY (2012) Different thermal tolerance and hsp gene expression in invasive and indigenous sibling species of *Bemisia tabaci* . Biol Invasions 14: 1587–1595.

[18] Lü ZC, Wan FH (2011) Using double-stranded RNA to explore the role of heat shock protein genes in heat tolerance in *Bemisia tabaci* (Gennadius). J Exp Biol 214: 764–789. 21307062 10.1242/jeb.047415

[19] Lü ZC, Wan FH (2008) Differential gene expression in whitefly (*Bemisia tabaci*) B-biotype females and males under heat-shock condition. Comp Biochem Physiol D 3: 257–262. 10.1016/j.cbd.2008.06.003 20494845

[20] Bowler K, Terblanche JS (2008) Insect thermal tolerance: what is the role of ontogeny, ageing and senescence. Biol Rev 83: 339–355. 18979595 10.1111/j.1469-185x.2008.00046.x

[21] Yu H, Wan FH (2009) Cloning and expression of heat shock protein genes in two whitefly species in response to thermal stress. J Appl Entomol 133: 602–614.

[22] Wang XW, Luan JB, Li JM, Bao YY, Zhang CX, et al (2010) De novo characterization of a whitefly transcriptome and analysis of its gene expression during development. BMC Genomics 11: 400–411. 20573269 10.1186/1471-2164-11-400 PMC2898760

[23] Wang XW, Luan JB, Li JM, Su YL, Xia J, et al (2011) Transcriptome analysis and comparison reveal divergence between two invasive whitefly cryptic species. BMC Genomics 12: 458–470. 21939539 10.1186/1471-2164-12-458 PMC3189941

[24] Wang XW, Zhao QY, Luan JB, Wang YJ, Yan GH, et al (2012) Analysis of a native whitefly transcriptome and its sequence divergence with two invasive whitefly species. BMC Genomics 13: 529–542. 23036081 10.1186/1471-2164-13-529 PMC3478168

[25] Pfaffl MW (2001) A new mathematical model for relative quantification in real-time RT-PCR. Nucleic Acids Res 29: e45. 11328886 10.1093/nar/29.9.e45 PMC55695

[26] Ghanim M, Kontsedalov S, Czosnek H (2007) Tissue-specific gene silencing by RNA interference in the whitefly *Bemisia tabaci* (Gennadius). Insect Biochem Mol 37: 732–738. 10.1016/j.ibmb.2007.04.006 17550829

[27] Miles PW (1965) Studies on the salivary physiology of plant-bugs: the saliva of aphids. J Insect Physiol 11: 1261–1268. 5828294 10.1016/0022-1910(65)90119-8

[28] Matsuura H, Sokabe T, Kohno K, Tominaga M, Kadowaki T (2009) Evolutionary conservation and changes in insect TRP Channels. BMC Evol Biol 9: 228–238. 19740447 10.1186/1471-2148-9-228 PMC2753570

[29] Duan B, Xu TL (2005) TRP channel and signal transduction. Acta Biophysica Sinica 21: 245–260.

[30] Han CY, Wang XL (2008) Recent advances on TRP channels. Prog Phys Sci 39: 27–32. 18357684

[31] Han Q (2009) Molecule mechanism of participation of TRP channel in temperature sensation. J Chengdu Med Coll 4: 220–224.

[32] Hamada FN, Rosenzweig M, Kang K, Pulver SR, Ghezzi A, et al (2008) An internal thermal sensor controlling temperature preference in *Drosophila* . Nature 454: 217–220. 18548007 10.1038/nature07001 PMC2730888

[33] Montell C (2005) *Drosophila* TRP channels. Pflugers Arch 451: 19–28. 15952038 10.1007/s00424-005-1426-2

[34] Damann N, Voets T, Nilius B (2008) TRPs in our senses. Curr Biol 18: R880–889. 18812089 10.1016/j.cub.2008.07.063

[35] Tracey WD, Wilson RI, Laurent G, Benzer S (2003) Painless, a *Drosophila* gene essential for nociception. Cell 113: 261–273. 12705873 10.1016/s0092-8674(03)00272-1

[36] Lee Y, Lee Y, Lee J, Bang S, Hyun S, et al (2005) Pyrexia is a new thermal transient receptor potential channel endowing tolerance to high temperatures in *Drosophila melanogaster* . Nat Genet 37: 305–310. 15731759 10.1038/ng1513

[37] Rosenzweig M, Brenman KM, Taylor TD, Phelps P, Patapoutian A, et al (2005) The *Drosophila* ortholog of vertebrate TRPA1 regulates thermotaxis. Genes Dev 19: 419–424. 15681611 10.1101/gad.1278205 PMC548941

[38] Karashima Y, Talavera K, Everaerts W, Janssens A, Kwan KY, et al (2009) TRPA1 acts as a cold sensor in vitro and in vivo. Proc Natl Acad Sci USA 106: 1273–1278. 19144922 10.1073/pnas.0808487106 PMC2633575

[39] Sokabe T, Tsujiuchi S, Kadowaki T, Tominaga M (2008) *Drosophila* painless is a Ca^2+^-requiring channel activated by noxious heat. J Neurosci 28: 9929–9938. 18829951 10.1523/JNEUROSCI.2757-08.2008 PMC6671277

[40] Neely GG, Keene AC, Duchek P, Chang EC, Wang QP, et al (2011) TrpA1 regulates thermal nociception in *Drosophila* . PLoS One 6: e24343. 21909389 10.1371/journal.pone.0024343 PMC3164203

[41] Kwon Y, Shim HS, Wang X, Montell C (2008) Control of thermotactic behavior via coupling of a TRP channel to a phospholipase C signaling cascade. Nat Neurosci 11: 871–873. 18660806 10.1038/nn.2170

[42] Sato A, Sokabe T, Kashio M, Yasukochi Y, Tominaga M, et al (2014) Embryonic thermosensitive TRPA1 determines transgenerational diapause phenotype of the silkworm, *Bombyx mori* . Proc Natl Acad Sci USA 111: E1249–E1255. 24639527 10.1073/pnas.1322134111 PMC3977306

